# Possible association of the *MTHFR* C677T polymorphism with familial Budd–Chiari syndrome in China: A case series

**DOI:** 10.1515/jtim-2026-0082

**Published:** 2026-10-23

**Authors:** Chen Li, Wanchen Yu, Yuhui Peng, Hong Guo, Haibin Su

**Affiliations:** Department of Severe Liver Diseases, Senior Department of Hepatology, the Fifth Medical Center of PLA General Hospital, Beijing, China; Department of Traditional Chinese Medicine, Beijing Rehabilitation Hospital of Capital Medical University, Beijing, China; Department of Medical Genetics, College of Basic Medical Science, Army Medical University, Chongqing, China

## To the editor

Budd–Chiari syndrome (BCS) is a rare but potentially fatal disease resulting from outflow tract obstruction of the hepatic vein and/or inferior vena cava (IVC). Inherited thrombophilia caused by gene mutations plays a significant role in the pathogenesis of BCS.^[1]^ However, the possible association between the *methylenetetrahydrofolate reductase* (*MTHFR*) gene C677T polymorphism and familial aggregation of BCS remains uncertain. We report the cases of three male siblings from rural regions of northern China who were diagnosed with chronic BCS. Two patients developed acute decompensation and organ failures on the basis of cirrhosis, leading to acute-on-chronic liver failure (ACLF). The two surviving siblings carry the *MTHFR* gene C677T TT genotype and have high levels of homocysteine and endothelin-1.

Case 1 concerns a 55-year-old male (Ⅱ.3 in Figure 1A). He developed abdominal distension at the age of 37 and followed by the gradual appearance of abdominal wall varices and intermittent abdominal pain. At the age of 54, he developed severe jaundice, prolonged prothrombin time, overt hepatic encephalopathy, and ACLF. Laboratory tests showed total bilirubin (TBIL) of 576.8 μmol/L, direct bilirubin (DBIL) of 410.6 μmol/L, alanine aminotransferase (ALT) of 86 U/ L, aspartate aminotransferase (AST) of 148 U/L, international normalized ratio (INR) of 1.33, blood ammonia of 88.4 μmol/L, and homocysteine of 30.6 μmol/L (Supplementary Table S1). Magnetic resonance imaging (MRI) revealed liver cirrhosis (LC), severe abdominal wall varices, and membranous obstruction of IVC (MOVC).^[2]^ He received portal pressure reduction, hepatocyte protection, folic acid supplementation (0.8 mg, once daily), and three sessions of artificial liver support system therapy, but he refused interventional procedures and liver transplantation. After receiving low-molecular-weight heparin (100 IU/kg, once per 12 h) for 1 week, he was switched to warfarin (2.5-3.0 mg, once daily) to maintain anticoagulant therapy and ensure that the target INR remained between 2 and 3. His liver function and abdominal wall varices improved within 2 months. After 6 months of treatment, by May 2025, his liver function and homocysteine levels had largely returned to normal (Supplementary Table S2). Meanwhile, Doppler ultrasonography showed improved patency of the IVC, and his Fibrosis-4 score decreased from 7.25 to 4.48.

**Figure 1:**
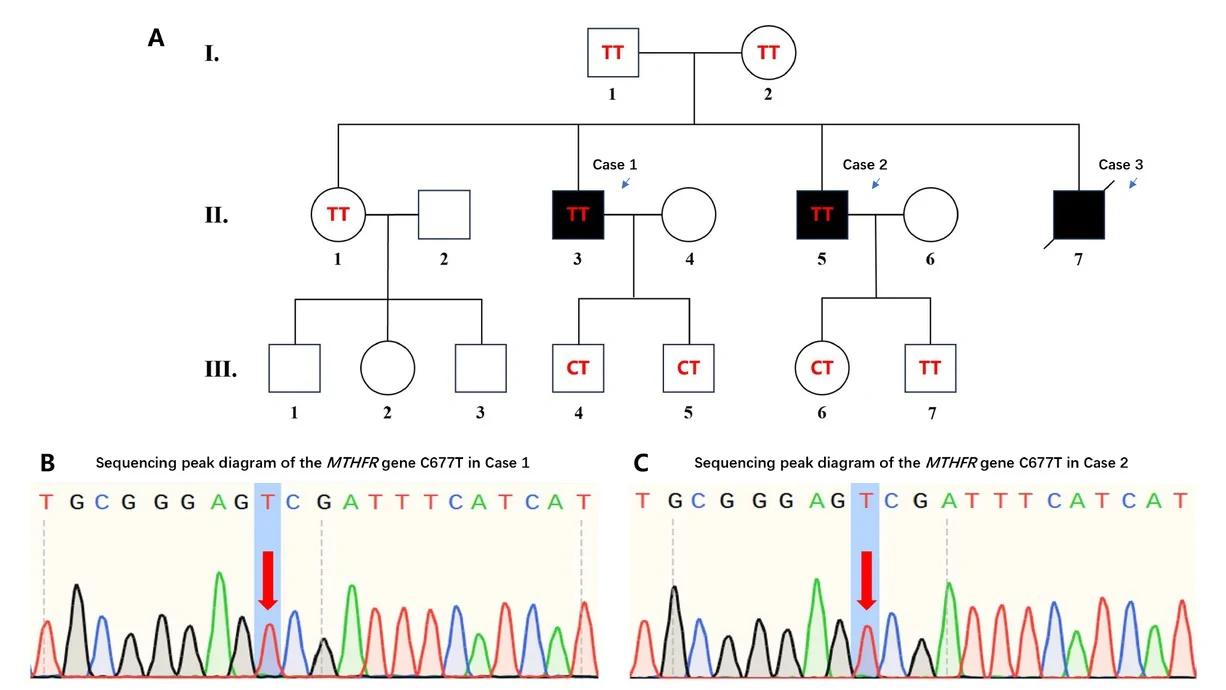
Family tree of BCS and results of MTHFR gene C677T polymorphism in this study. (A) Circles and squares indicate female and male family members, respectively. Black and white symbols indicate family members with and without BCS, respectively. A strikethrough line indicates a deceased individual. Characters enclosed in boxes denote the MTHFR gene C677T genotype. TT and CT represent the MTHFR gene C677T TT and CT genotypes, respectively. No characters appear in a box when the individual did not undergo genetic testing. (B) Sequencing peak diagram of the MTHFR gene C677T in Case 1. The red arrow indicates the presence of the TT variant in the MTHFR gene C677T. (C) Sequencing peak diagram of the MTHFR gene C677T in Case 2. The red arrow indicates the presence of the TT variant in the MTHFR gene C677T. BCS: Budd–Chiari syndrome; MTHFR: methylenetetrahydrofolate reductase.

Case 2 concerns a 53-year-old male (Ⅱ.5 in Figure 1A). At the age of 43, he experienced abdominal distension and abdominal wall varices. At the age of 44, he was diagnosed with BCS and LC caused by MOVC after undergoing MRI and IVC venography. Laboratory tests showed TBIL of 20.4 μmol/L, DBIL of 9.8 μmol/L, ALT of 34 U/ L, AST of 26 U/ L, and homocysteine of 26.2 μmol/L (Supplementary Table S1).

He underwent his first balloon angioplasty of the IVC and received continuous anticoagulation with warfarin (2.5-3.0 mg, once daily) after the procedure, maintaining his INR within the range of 2.0-3.0. Five years later, he underwent a second balloon angioplasty of the IVC owing to the recurrence of IVC stenosis. Six months later, he underwent three sessions of injection sclerotherapy for esophageal varices due to acute upper gastrointestinal bleeding caused by portal hypertension. Eleven months later, he underwent a third balloon angioplasty and stenting of the IVC for recurrent IVC stenosis. Afterward, he received hepatocyte protection, anticoagulation, and folic acid supplementation (0.8 mg, once daily). Over the past 3 years, endoscopy has confirmed that his portal hypertension has improved, and MRI has shown no recurrence of IVC stenosis. In May 2025, his liver function and homocysteine levels were normal (Supplementary Table S2).

Case 3 concerns a 49-year-old male (Ⅱ.7 in Figure 1A). At the age of 29, he developed abdominal distension and abdominal wall varices, which were diagnosed as BCS by Doppler ultrasonography. He did not receive any treatment until the age of 48, when MRI revealed the presence of LC and hepatocellular carcinoma (HCC). Despite two transcatheter arterial chemoembolization procedures for advanced HCC, his condition continued to progress. Ten months later, he developed ACLF and acute kidney injury. Laboratory tests showed TBIL of 429.8 μmol/L, DBIL of 332.8 μmol/L, ALT of 44 U/L, AST of 107 U/L, creatinine of 167 μmol/L, homocysteine of 28.7 μmol/L, INR of 1.35, and alpha-fetoprotein of 553 μg/L (Supplementary Table S1). MRI revealed LC, severe abdominal wall varices, MOVC, and HCC.^[2]^ The patient declined active treatment and ultimately died of multiple organ failure 2 months later (Supplementary Table S2).

Whole-exome sequencing revealed that Cases 1 and 2, as well as their parents (Ⅰ.1 and Ⅰ.2 in Figure 1A), possessed the *MTHFR* 677TT genotype (Figure 1A-C). However, no factor V Leiden, prothrombin G20210A, calreticulin, or Janus kinase 2 V617F gene mutations were detected. Doppler ultrasonography confirmed that the other family members did not have BCS. The endothelin-1 levels of Cases 1 and 2 were higher than the upper limit of normal, whereas those of the other family members were within the normal range. Compared with the three family members with the CT genotype, the six family members with the TT genotype exhibited a potential trend toward lower levels of the MTHFR enzyme and folic acid, as well as higher levels of endothelin-1 and homocysteine (Supplementary Table S3).

BCS exhibits a certain degree of familial aggregation, with similar types and locations of obstruction and clinical manifestations.^[3]^ In this study, the two living patients and their parents all carried the *MTHFR* 677TT genotype. From a genetic perspective, we speculate that the deceased sibling in Case 3 may also have possessed this specific genotype. Compared with other types of BCS, MOVC typically presents with a gradual onset, abdominal pain, and large collateral vessels in the anterior and posterior regions of the trunk. MOVC may progress to congestive fibrosis and cirrhosis, leading to persistent hepatocellular necrosis, which is closely associated with the development of HCC.^[4]^

Increasing evidence suggests that the *MTHFR* gene C677T polymorphism and hyperhomocysteinemia (HHcy) are closely associated with BCS.^[1]^ Variations at position 677 in the *MTHFR* gene lead to reduced MTHFR enzyme activity, which affects methionine and folate metabolism, resulting in excessive homocysteine formation and lower folate levels. This is particularly evident in males and those residing in rural areas when folic acid supplementation is lacking.^[5]^ Compared with the CC genotype, the TT genotype has higher homocysteine levels, and the CT genotype has mildly elevated homocysteine levels. HHcy can directly increase endothelin-1 levels in the blood or indirectly elevate them by upregulating endothelin receptors in vascular smooth muscle cells, ultimately leading to vascular endothelial damage.^[6]^ HHcy has the potential to further disrupt anticoagulant and fibrinolytic systems. This may be the primary mechanism by which *MTHFR* gene C677T variants trigger a series of venous thromboembolic events, including BCS.

Six family members were found to carry the *MTHFR* 677TT genotype, but only two males from the second generation developed the disease. The *MTHFR* gene C677T polymorphism may be a contributory factor to genetic susceptibility to BCS. However, the genetic background of BCS may involve the combined effects of multiple genes rather than a single strongly pathogenic gene.^[7]^ Furthermore, due to the potential presence of incomplete penetrance, family members who carry the specific genotype may not all develop BCS.

Prompt identification of BCS is crucial; upon diagnosis, treatment should be initiated according to disease severity and rate of progression. The 10-year cumulative survival rate for patients who receive treatment (75.2%) is significantly higher than that for patients who do not (53.1%).^[8]^ Anticoagulation, treatment of underlying diseases, and management of the complications of portal hypertension should be considered basic therapeutic measures. Angioplasty, stenting, and transjugular intrahepatic portosystemic shunt are considered further treatment options for patients with poor medical response, and liver transplantation is regarded as the last resort. For MOVC, angioplasty alone is effective, but routine stenting combined with angioplasty is superior in preventing restenosis.^[9]^ Only 5.0% of patients with BCS present as ACLF, but they have a high mortality. Appropriate interventional procedures can facilitate recovery from liver failure and severe jaundice.^[10]^

In conclusion, we report three siblings of BCS with the MOVC type and similar clinical manifestations. Both surviving patients carry the *MTHFR* 677TT genotype, and this variant may be associated with familial aggregation of BCS.

## Supplementary Information

Supplementary materials are only available at the official site of the journal (www.intern-med.com).

## Supplementary Material

Supplementary Material Details
