# Supplementary material for: Possible association of the MTHFR C677T polymorphism with familial Budd–Chiari syndrome in China: A case series
Source: J Transl Int Med. 2026 Oct 23;14(5):838–41. doi: 10.1515/jtim-2026-0082 (PMC13726656; doi:10.1515/jtim-2026-0082)
Supplement: Supplementary file 1 [file jtim-2026-0082_sm.pdf]

Supplementary materials

Supplementary Table S1: Clinical manifestations of these three patients

**Clinical features**	**Case 1**	**Case 2**	**Case 3**	**Normal range**
Gender	Male	Male	Male	N/A
Family relation-ship	Eldest brother	Second brother	Youngest brother	N/A
Career	Farmer	Rural civil serv-ant	Farmer	N/A
Symptomatic age (years)	37	43	29	N/A
Treatment age (years)	54	44	48	N/A
Initial symptom	Abdominal pain	Abdominal dis-tension	Abdominal dis-tension	N/A
Abdominal wall varices	Existent	Existent	Existent	Inexistent
Jaundice	Existent	Inexistent	Existent	Inexistent
Liver cirrhosis	Existent	Existent	Existent	Inexistent
HCC	Inexistent	Inexistent	Existent	Inexistent
HE	Existent	Inexistent	Inexistent	Inexistent
AKI	Inexistent	Inexistent	Existent	Inexistent
ACLF	Existent	Inexistent	Existent	Inexistent
Obstruction type	MOVC	MOVC	MOVC	Inexistent
TBIL (μmol/L)	576.8	20.4	429.8	0-26
DBIL (μmol/L)	410.4	9.8	332.8	0-4
ALT (U/L)	86	34	44	9-50
AST (U/L)	148	26	107	15-40
ALP (U/L)	93	101	106	45-125

GGT (U/L)	88	71	34	10-60
INR	1.33	1.23	1.35	0.8-1.2
BLA (μmol/L)	88.4	Not done	51.3	0-30
Cre (μmol/L)	65	71	167	57-97
Homocysteine (μmol/L)	30.6	26.2	28.7	5-15
WBCs ($\times 10^9$/L)	6.11	6.50	2.89	3.5-9.5
HGB (g/L)	131	153	78	130-175
PLTs ($\times 10^9$/L)	121	68	40	125-350
Antithrombin III (%)	47.0	Not done	62.7	75-125
HBsAg	Negative	Negative	Negative	Negative
Anti-HCV	Negative	Negative	Negative	Negative
APLA	Negative	Negative	Negative	Negative
ANA	Negative	Negative	Negative	Negative
AMA	Negative	Negative	Negative	Negative
Bone marrow	Normal state	Normal state	Not done	Normal state
CD55 expression	Normal state	Normal state	Not done	Normal state
MTHFR gene C677T polymorphism	TT genotype	TT genotype	Not done	N/A
ALSS	Existent	Inexistent	Inexistent	N/A
Anticoagulation	Existent	Existent	Inexistent	N/A
Interventional procedures	Inexistent	Existent	Inexistent	N/A
Liver transplantation	Inexistent	Inexistent	Inexistent	N/A
Outcomes	Alive	Alive	Dead	N/A

HCC: hepatocellular carcinoma; HE: hepatic encephalopathy; AKI: acute kidney injury; ACLF: acute-on-chronic liver failure; MOVC: membranous obstruction of inferior vena

cava; TBIL: total bilirubin; DBIL: direct bilirubin; ALT: alanine aminotransferase; AST: aspartate aminotransferase; ALP: alkaline phosphatase; GGT: glutamyltransferase; INR: international normalized ratio; BLA: blood ammonia; Cre: creatinine; WBCs: white blood cells; HGB: hemoglobin; PLTs: platelets; HBsAg: Hepatitis B surface antigen; Anti-HCV: antibody of hepatitis C virus; APLA: antiphospholipid antibodies; ANA: antinuclear antibodies; AMA: antimitochondrial antibodies; MTHFR: methylenetetrahydrofolate reductase; ALSS: artificial liver support system; N/A: not applicable.

Supplementary Table S2: Interventions and outcomes of key events for these three patients

Patient	Time	Key events	Interventions	Outcomes
	2007.05	Discomfort occurred	Untreated	Disease progression
	2024.10-2024.11	Was diagnosed with BCS-ACLF and LC	Portal pressure reduction, hepatocyte protection, folic acid supplementation, ALSS, anticoagulation	Condition improved
Case 1	2025.05	Follow-up	Portal pressure reduction, hepatocyte protection, folic acid supplementation, anticoagulation	TBIL 38.8 μmol/L, DBIL 20.3 μmol/L, ALT 35 U/L, AST 26 U/L, homocysteine 15.5 μmol/L, INR 2.2
	2026.05	Follow-up	Portal pressure reduction, hepatocyte protection, folic acid supplementation, anticoagulation	Stable condition

Case 2	2015.01	Discomfort occurred	Untreated	Disease progression
	2016.01	Was diagnosed with BCS and LC	Balloon angioplasty of the IVC	Condition improved
	2021.04	Recurrence of IVC stenosis	Balloon angioplasty of the IVC	Condition improved
	2021.10	Acute upper gastrointestinal bleeding	Injection sclerotherapy for esophageal varices	Condition improved
	2022.09	Recurrence of IVC stenosis	Balloon angioplasty and stenting of the IVC	Condition improved
	2025.05	Follow-up	Hepatocyte protection, anticoagulation, folic acid supplementation	TBIL 19.8 μmol/L, ALT 30.4 U/L, AST 28.7 U/L, homocysteine 13.2 μmol/L, INR 2.3
	2026.05	Follow-up	Hepatocyte protection, anticoagulation, folic acid supplementation	Stable condition
Case 3	2004.07	Experienced discomfort and was diagnosed with BCS	Untreated	Disease progression
	2023.08-2023.11	Was diagnosed with LC and HCC	Transcatheter arterial chemoembolization	Disease progression
	2024.09-2024.10	Was diagnosed with BCS-ACLF	Hepatocyte protection	Disease progression

	and HCC		
2024.12	Follow-up	Gave up treatment	Died of multiple organ failure

BCS: Budd–Chiari Syndrome; ACLF: acute-on-chronic liver failure; LC: liver cirrhosis; ALSS: artificial liver support system; IVC: inferior vena cava; TBIL: total bilirubin; DBIL: direct bilirubin; ALT: alanine aminotransferase; AST: aspartate aminotransferase; INR: international normalized ratio; HCC: hepatocellular carcinoma.

Supplementary Table S3: The levels of MTHFR enzyme, homocysteine, endothelin-1, and folic acid in this family lineage

**Family members**	***MTHFR* gene C677T polymorphism**	**MTHFR enzyme (U/L) (Normal range: 100-800)**	**Homocysteine (ng/mL) (Normal range: 5-40)**	**Endothelin-1 (pg/mL) (Normal range: 20-160)**	**Folic acid (pg/mL) (Normal range: 150-1200)**
Ⅰ.1	TT genotype	475.7	32.4	149.5	533.4
Ⅰ.2	TT genotype	419.6	31.1	144.6	542.5
Ⅱ.1	TT genotype	407.7	28.5	127.8	470.1
Ⅱ.3 (Case 1)	TT genotype	502.2	35.4	178.8	561.7
Ⅱ.5 (Case 2)	TT genotype	380.1	37.3	186.7	491.0
Ⅲ.4	CT genotype	676.0	22.2	73.1	813.5
Ⅲ.5	CT genotype	748.8	24.1	89.6	884.7
Ⅲ.6	CT genotype	656.8	24.5	99.7	917.8
Ⅲ.7	TT genotype	297.5	30.1	123.5	663.5
CT genotype group (n = 3)	CT genotype	693.9 ± 48.5	23.6 ± 1.2	87.5 ± 13.4	872.0 ± 53.3

TT genotype group (n = 6)	TT genotype	413.8 ± 72.6	32.5 ± 3.3	151.8 ± 26.0	543.7 ± 67.8
t	N/A	-5.943	4.352	3.936	-7.256
P	N/A	0.001	0.003	0.006	< 0.001

P: Comparison between CT genotype and TT genotype groups; *MTHFR*: *methylene-tetrahydrofolate reductase*; N/A: not applicable.
